# Biohydrogen Production by the Thermophilic Bacterium *Caldicellulosiruptor saccharolyticus*: Current Status and Perspectives

**DOI:** 10.3390/life3010052

**Published:** 2013-01-17

**Authors:** Abraham A. M. Bielen, Marcel R. A. Verhaart, John van der Oost, Servé W. M. Kengen

**Affiliations:** Laboratory of Microbiology, Wageningen University, Dreijenplein 10, 6703 HB Wageningen, The Netherlands; E-Mails: verhaart@biogast.nl (M.R.A.V.); john.vanderoost@wur.nl (J.O.); serve.kengen@wur.nl (S.W.M.K.)

**Keywords:** *Caldicellulosiruptor saccharolyticus*, biohydrogen, dark fermentation, cellulolytic thermophile, thermodynamics, rhamnose metabolism, pyrophosphate, redox balance, hydrogen inhibition, regulation

## Abstract

*Caldicellulosiruptor saccharolyticus* is one of the most thermophilic cellulolytic organisms known to date. This Gram-positive anaerobic bacterium ferments a broad spectrum of mono-, di- and polysaccharides to mainly acetate, CO_2_ and hydrogen. With hydrogen yields approaching the theoretical limit for dark fermentation of 4 mol hydrogen per mol hexose, this organism has proven itself to be an excellent candidate for biological hydrogen production. This review provides an overview of the research on *C. saccharolyticus* with respect to the hydrolytic capability, sugar metabolism, hydrogen formation, mechanisms involved in hydrogen inhibition, and the regulation of the redox and carbon metabolism. Analysis of currently available fermentation data reveal decreased hydrogen yields under non-ideal cultivation conditions, which are mainly associated with the accumulation of hydrogen in the liquid phase. Thermodynamic considerations concerning the reactions involved in hydrogen formation are discussed with respect to the dissolved hydrogen concentration. Novel cultivation data demonstrate the sensitivity of *C. saccharolyticus* to increased hydrogen levels regarding substrate load and nitrogen limitation. In addition, special attention is given to the rhamnose metabolism, which represents an unusual type of redox balancing. Finally, several approaches are suggested to improve biohydrogen production by *C. saccharolyticus*.

## 1. Introduction

The use of renewable plant biomass for the production of biofuels, chemicals or other biocommodities can provide a realistic alternative for fossil fuel based processes [1,2]. The implementation of lignocellulosic biomass for biofuel production requires the degradation of recalcitrant substrates like cellulose, hemicellulose or lignin. Lignin is either removed or modified [3], while cellulose and hemicellulose are converted into more readily fermentable mono-, di- and oligo-saccharides. Although this can be achieved by different (thermo)chemical or enzymatic pre-treatments, a more desirable process combines both substrate hydrolysis and fermentation of complex plant biomass. Such a “consolidated bioprocess” (CBP) circumvents the negative environmental impact inherent to (thermo)chemical pre-treatment and might limit overall process costs [1,4].

Hydrogen gas (H_2_) is considered an alternative for the non-renewable fossil fuels and can be produced in a carbon neutral process. The controlled biological production of H_2_ would allow for capturing the CO_2_ released during the process, preventing it to dissipate into the environment. In addition, compared to carbon based (bio)fuel types, H_2_ has the advantage that (i) during its oxidation only H_2_O is released and (ii) that H_2_ fuel cells can be used, which are more energy efficient than the presently used combustion engines [5]. Biohydrogen can be produced from renewable feedstocks in an anaerobic fermentation process, which is often referred to as dark fermentation to distinguish it from photofermentative hydrogen production.

Both plant biomass degradation and biological H_2_ formation appear advantageous under thermophilic conditions. Moreover, thermophiles display an extensive glycoside hydrolase inventory aiding in the lignocellulosic biomass breakdown [6,7,8]. Based on thermodynamic considerations H_2_ formation is more feasible at elevated temperatures [9,10]. Correspondingly, H_2_ yields are in general higher for (hyper)thermophiles, reaching the theoretical limit of 4 mol H_2_ per mol of hexose, compared to the mesophilic hydrogen producers [9,11,12].

Since its isolation in the mid-eighties it has become clear that the thermophilic anaerobic bacterium *Caldicellulosiruptor saccharolyticus* [13] displays both the desirable polysaccharide degrading capabilities (including cellulose) and H_2_ producing characteristics, making it an outstanding source for thermostable glycoside hydrolases and an excellent candidate for biohydrogen production from renewable biomass.

This review will discuss the available scientific data on *C. saccharolyticus* regarding its lignocellulolytic capability, substrate specificity, catabolism and H_2_ producing capacity, which has made *C. saccharolyticus* to become a model organism for the study of fermentative hydrogen formation at elevated temperatures.

## 2. Isolation and Initial Characterization

The foreseen commercial value of thermostable cellulolytic enzymes in biotechnological applications triggered the investigation of new sources of these types of enzymes. In the search for novel thermophilic cellulolytic micro-organisms, several anaerobic bacteria have been isolated from natural enrichment sites from the Rotorua-Taupo thermal area in New Zealand [14]. One of the isolated strains, TP8.T 6331 [14] also referred to as TP8 [15] or “*Caldocellum saccharolyticum*” [16], revealed thermostable cellulase activity up to 85 °C [14,15] but also lignocellulolytic biomass decomposition capabilities [16]. Strain TP8.T 6331 was assigned to a new genus *Caldicellulosiruptor* as *Caldicellulosiruptor saccharolyticus* and was characterized as a Gram-positive, asporogenous, extremely thermophilic and strictly anaerobic bacterium capable of sustaining growth at a temperature range of 45–80 °C (T_opt_ =70 °C) and pH range of 5.5–8.0 (pH_opt_ = 7) [13]. Acid production could be detected for a broad substrate range including different pentoses and hexoses, di-saccharides and polysaccharides like cellulose and xylan [13,14,15,16]. In particular, the capacity to use cellulose at high temperatures was exceptional.

Ever since, several cellulolytic and weakly cellulolytic *Caldicellulosiruptor* species have been identified, all of which are isolated from terrestrial geothermal regions. [13,14,17,18,19,20,21,22,23,24,25,26,27,28]. The availability of the fully sequenced genomes of 8 of these *Caldicellulosiruptor* species allows the investigation of the possible differences in their cellulolytic traits and the analysis of other remarkable features of this genus [29,30,31,32,33].

## 3. Hydrolytic Capacity and Complex Biomass Decomposition

For the decomposition of recalcitrant plant polysaccharides *C. saccharolyticus* does not employ cellulosome-like structures, as described for some *Clostridium* species [34], but wields a variety of free-acting endo- and exo-glycoside hydrolases (GH) capable of hydrolyzing the glycosidic bonds of α- and β-glucans like starch, pullulan and cellulose, but also xylan and hetero-polysaccharides like hemicelluloses and pectin [8,13,33,35,36]. Actually, *Caldicellulosiruptor* species, together with *Thermoanaerobacter* species, are one of the most thermophilic crystalline cellulose-degrading organisms known to date that use free-acting primary cellulases [7]. *C. saccharolyticus* contains 59 open reading frames (ORFs) that include GH catalytic domains [29]. Some of these ORFs code for multifunctional, multi-domain proteins that contain glycoside hydrolase domains, belonging to different GH families, and multiple carbon binding modules [6,29,36]. The catalytic properties and structural organization of some of the glycoside hydrolases from *C. saccharolyticus*, have been extensively investigated.

A β-glucosidase (BglA) [37,38], β-xylosidase [39], β-1,4-xylanase [40] and a type I pullulanase [41] from *C. saccharolyticus* have been cloned into *E. coli* and characterized. The majority of the genes encoding xylan degradation associated enzymes appear to be clustered on the genome (*xynB-xynF*, Csac_2404-2411) [6,36]. Both XynA and XynE exhibit endoxylanase and xylosidase activity [36,42], while XynB only acts as a β-D-xylosidase [43]. XynC does not contain a GH domain and was shown to be an acetyl esterase [44], XynD showed to be active on xylan [36] and although the cloning and expression of intact multi-domain XynF could not be achieved, its *N*- and *C*-terminal parts revealed catalytic activity on arabinoxylan. Hence XynF was proposed to be involved in the degradation of the arabinoxylan component of hemicellulose [36]. A second locus covers several genes coding for multidomain proteins involved in glucan and mannan hydrolysis (*celA-manB*, Csac_1076-1080) [6,36]. *CelA* is coding for a multidomain cellulase [45], the bifunctional cellulase CelB exhibited both endo-β-1,4-glucanase and exo-β-1,4-glucanase activity [36,46,47] and CelC was characterized as an endo-1,4-β-D-glucanase [48]. ManA was characterized as a β-mannanase [49], but *ManB* codes for an inactive mannase, which after correcting for a frame shift in the nucleotide sequence, exhibited β-mannanase activity [48]. Several ORFs of the described *celA-manB* and *xynB-xynF* loci were differentially transcribed on pretreated poplar and switch grass compared to the monosaccharides glucose and xylose, showing their involvement in the decomposition of complex carbohydrates [36].

While some of the GH proteins act intracellularly, others are excreted, allowing the decomposition of non-soluble substrates to smaller oligo- or mono-saccharides. Early findings by Reynolds *et al*. already indicated that a significant percentage of the cellulolytic activity was found to be associated with insoluble substrate [15]. These interactions, between GH and substrate, are facilitated by carbon binding modules (CBM). CBMs allow the positioning of the GH catalytic domains in the vicinity of the substrate, thus increasing the rate of catalysis. Interestingly, most multi-domain GHs identified in *C. saccharolyticus*, containing one or more CBMs, possess a signal peptide, which mark them for excretion [36]. A relative higher amount of GH related proteins could be observed in the substrate bound protein fraction, from *Caldicellulosiruptor* species grown on Avicel, with respect to the whole cell proteome [29]. Additionally, these proteome studies allowed the identification of specific GHs which interact with crystalline cellulose. CelA, a multi-domain GH consisting of two GH domains (GH9 and GH48) and three CBM3 modules, was found to be the most abundant substrate bound protein for strong cellulolytic *Caldicellulosiruptor* species [29]. The sequenced genomes of *Caldicellulosiruptor* species reveal differences in glycoside hydrolytic capacity, which reflects their difference in biomass degrading capabilities [29,35]. The secretome of *C. saccharolyticus* grown on glucose contains several carbohydrate-degrading enzymes including CelA, ManA, CelB and CelC (protein sequence ID A4XIF5/6/7/8 respectively), which indicates that these GH are constitutively expressed even under non-cellulose degrading conditions [50].

In addition to the interactions between substrate and glycoside hydrolases, interactions between whole cells and a substrate have also been observed for *C. saccharolyticus.* These interactionsappeared to be substrate specific. For instance, a higher degree of cell-to-substrate attachment was observed for cells grown on switch grass, compared to poplar [36]. Several S-layer homology (SLH) domain containing proteins, which have been identified in *Caldicellulosiruptor* species, are proposed to have a role in such cell substrate interactions. These SLH domain proteins contain both glycoside hydrolases domains and non-catalytic carbohydrate binding domains, which are utilized in lignocellulose degradation by both recruiting and degrading complex biomass via cell substrate interactions [51]. In addition, cell immobilization on a support matrix, like pine wood shavings, supports cell survival and improves the H_2_ evolving capacity of *C. saccharolyticus* [52].

## 4. Sugar Catabolism and Pathway Regulation

### 4.1. Sugar Uptake and Fermentation

**Figure 1:**
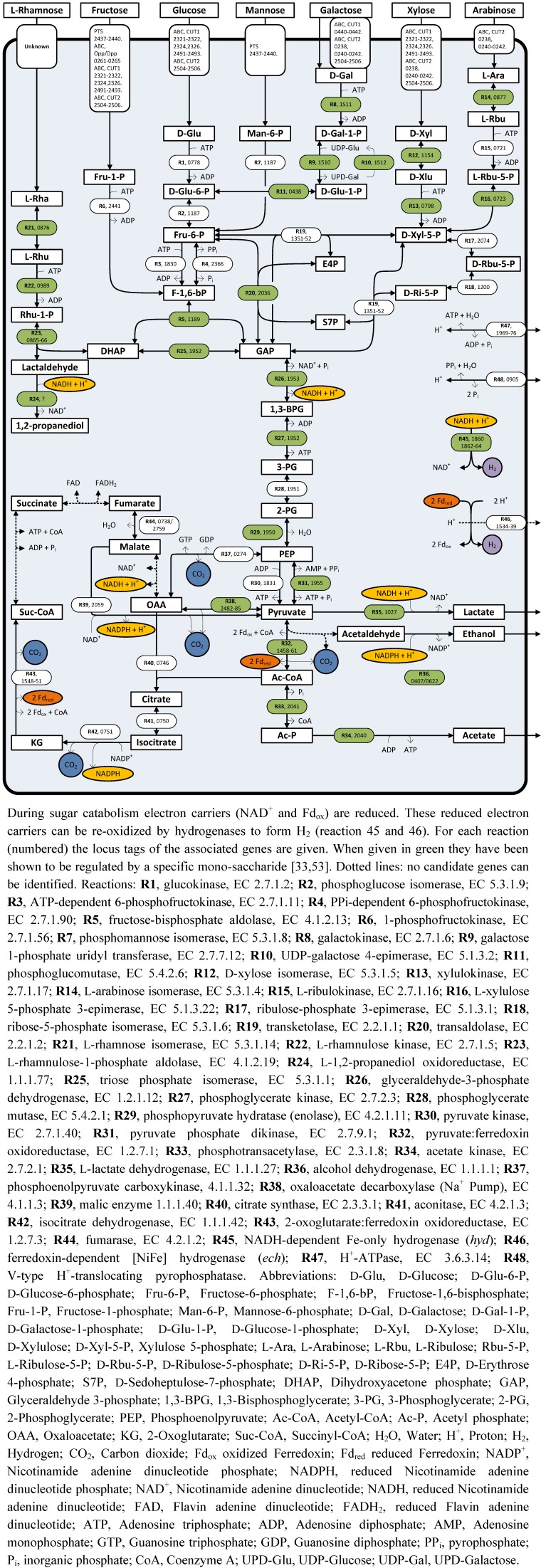
Overview of the central carbon metabolism of *Caldicellulosiruptor saccharolyticus*.

Soluble sugar substrates can enter *C. saccharolyticus* cells either as mono-, di- or oligo-saccharides via several ABC-transporters, which facilitate substrate transport across the membrane at the expense of ATP. In addition, *C. saccharolyticus* contains one fructose specific phosphotransferase system (PTS). During PTS-mediated transport the substrate is both transported and phosphorylated at the expense of phosphoenolpyruvate (PEP). The substrate specificity of the 24 sugar ABC transport systems, identified in *C. saccharolyticus*, has been assigned based on bioinformatic analysis and functional genomics [53]. Most of the identified ABC transporters have a broad predicted substrate specificity and for some transporters the annotated substrate specificity was confirmed by transcriptional data obtained from cells grown on different mono-saccharides [53]. Some substrates can be transported by multiple transporter systems (Figure 1).

The growth of *C. saccharolyticus* on sugar mixtures revealed the co-utilization of hexoses and pentoses, without any signs of carbon catabolite repression (CCR) [33,53]. The absence of CCR is in principle a very advantageous characteristic of *C. saccharolyticus*, as it enables the simultaneous fermentation of hexoses and pentoses [33]. Substrate co-utilization has also been confirmed for biomass derived hydrolysates [54,55]. Despite the absence of CCR, a somewhat higher preference for the pentose sugars (xylose and arabinose) with respect to the hexose sugars (glucose, mannose and galactose) was demonstrated, but the highest preference was observed for the PTS-transported hexose fructose [53].

Once inside the cell, the sugar substrates are converted into a glycolytic intermediate. NMR analysis of the fermentation end-products of *C. saccharolyticus* grown on ^13^C-labeled glucose showed that the Embden-Meyerhof pathway is the main route for glycolysis [56]. All genes encoding components of the Embden-Meyerhof and non-oxidative pentose phosphate pathway have been identified in the genome. There is no evidence for the presence of the oxidative branch of the pentose phosphate pathway or the Entner-Doudoroff pathway [33] (Figure 1).

Each sugar substrate, with the exception of rhamnose is completely catabolized to glyceraldehyde 3-phosphate (GAP) (rhamnose catabolism will be discussed separately in more detail below). The subsequent conversion of GAP to pyruvate, via the C-3 part of glycolysis, results in the formation of the reduced electron carrier NADH. Pyruvate can be further oxidized to acetyl-CoA by pyruvate: ferredoxin oxidoreductase (POR), which is coupled to the generation of reduced ferredoxin (Fd_red_). Finally, acetyl-CoA can be converted to the fermentation end-product acetate (Figure 1).

Both types of reduced electron carriers (NADH and Fd_red_) can be used by hydrogenases for proton reduction, thus forming H_2_. The genome of *C. saccharolyticus* contains a gene cluster coding for an NADH-dependent cytosolic hetero-tetrameric Fe-only hydrogenase (*hyd*) and a cluster encoding a membrane bound multimeric [NiFe] hydrogenase (*ech*), which presumably couples the oxidation of Fd_red_ to H_2_ production [33]. Under optimal cultivation conditions, when all reductants are used for H_2_ formation, the complete oxidation of glucose yields 4 mol of H_2_ per mol of glucose consumed (Equation 1).

glucose + 4·H_2_O → 2 acetate^−^ + 2·HCO_3_^−^ + 4·H^+^ + 4·H_2_ (1)

glucose + 2·H_2_O → 2 ethanol + 2·HCO_3_^-^ + 2·H^+^ (2)

glucose → 2 lactate^-^ + 2·H^+^ (3)

However, suboptimal growth conditions lead to a mixed acid fermentation with ethanol (Equation 2) and lactate (Equation 3) as end products in addition to acetate and H_2_. Lactate is produced from pyruvate, using NADH as electron donor and catalyzed by lactate dehydrogenase (LDH). The corresponding *ldh* gene could be easily identified in the genome [33,57]. However, the identity of the enzymes and genes involved in ethanol formation, are less clear. Two alcohol dehydrogenase (ADH) genes have been identified in the genome which, based on transcriptional data, can both be involved in ethanol formation from acetaldehyde. The way acetaldehyde is produced, is however, not known. Acetaldehyde can be produced from pyruvate by a pyruvate decarboxylase, as described for yeast, or from acetyl-CoA by an acetaldehyde dehydrogenase, as is commonly seen in fermentative bacteria. In several thermophilic ethanol-producing bacteria, acetaldehyde is produced by a bifunctional acetaldehyde/ethanol dehydrogenase [58,59]. However, no candidate gene could be identified for any of these alternatives [33,60]. A third option might be that acetaldehyde is formed from pyruvate in a CoA-dependent side reaction of the pyruvate:ferredoxin oxidoreductase as described for *Pyrococcus furiosus* [61]. There is no experimental evidence for such a side reaction in *C. saccharolyticus*, but the absence of a dedicated enzymatic acetaldehyde-forming step might explain the low ethanologenic capacity of *C. saccharolyticus*. The difference in the observed lower ethanol to acetate ratio for *C. saccharolyticus* with respect to *Clostridium thermocellum* [16] can be explained by the fact that the *C. saccharolyticus* genome does not have a similar pathway for ethanol formation as identified in *C. thermocellum* [58]. Similar to other high yield ethanol-producing thermophiles, like *Thermoanaerobacter ethanolicus* [62] or *Thermoanaerobacterium saccharolyticum* [63], *Clostridium thermocellum* has a bifunctional acetaldehyde-CoA/alcohol dehydrogenase, which catalyzes ethanol formation from acetyl-coA [58,59]. Such a pathway is absent in low level ethanol-producing thermophiles like *C. saccharolyticus* [33], *Thermoanaerobacter tengcongensis* [64] and *Pyrococcus furiosus* [65]. Although *C. saccharolyticus* is able to produce some ethanol, the flux through the ethanol-forming pathway is apparently limited resulting in the lower ethanol to acetate ratio.

NADPH is assumed to be the preferred substrate for the ethanol-forming ADH reaction [33,57]. A potential source of NAPDH could be the isocitrate dehydrogenase in the oxidative branch of the incomplete TCA cycle (Figure 1). Alternatively, NADPH can be produced from NADH, but so far no candidate genes coding for such transhydrogenase have been identified in *C. saccharolyticus* [33,60]. With respect to H_2_ production, it is important to realize that only the production of acetate is coupled to H_2_ formation since no net reducing power remains for H_2_ formation when ethanol or lactate is produced.

### 4.2. Rhamnose Fermentation

**Figure 2:**
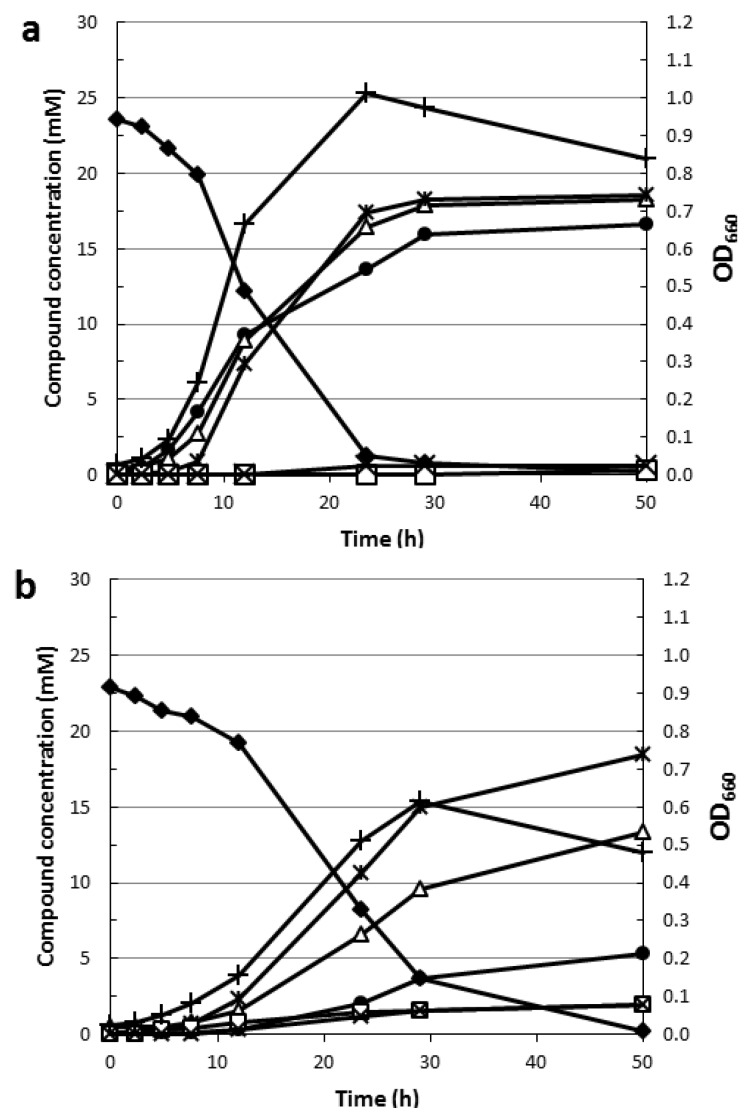
Fermentation profile of *C. saccharolyticus* grown on rhamnose batch cultivation: (**a**) without CO in the headspace (**b**) with a 100% CO headspace. Rhamnose (diamonds), acetate (open triangles), 1,2 propanediol (asterisks), lactate (open squares), ethanol (crosses), H_2_ (circles) and OD_660_ (plus sign).

**Figure 3:**
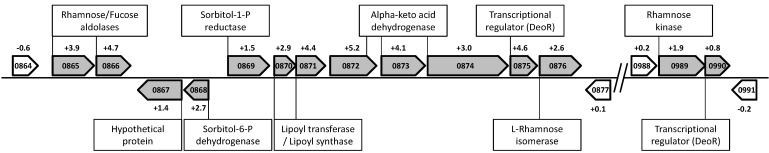
Schematic representation of two rhamnose associated gene clusters from *C. saccharolyticus* (Csac_0865-Csac_0876 and Csac_0989-Csac_0990). For each member of the cluster (grey arrows) the proposed function (text box) and locus tag number (four digit number) is given. Presented log2 values represent the ratio between transcription levels of the specific gene during growth on rhamnose with respect to growth on glucose; (+) upregulated, (−) downregulated on rhamnose versus glucose [33].

Compared to glucose, fermentative growth on the deoxy sugar rhamnose is associated with a different carbon and electron metabolism. The proposed pathway for rhamnose degradation (Figure 1) implies that during rhamnose catabolism half of the generated reduced electron-carriers is used for the reduction of lactaldehyde to 1,2-propanediol, while the other half can be recycled through H_2_ formation [33]. Indeed, fermentation of rhamnose by *C. saccharolyticus* results in the production of 1,2-propanediol, acetate, H_2_ and CO_2_ in a 1:1:1:1 ratio ([66], Figure 2a). This ratio suggests that all NADH is used for 1,2-propanediol formation and that all Fd_red_ is used for H_2_ formation. However, when *C. saccharolyticus* is grown on rhamnose under a headspace of carbon monoxide (CO), which is an established competitive inhibitor of both NiFe- [67] and Fe-only [68] hydrogenases, H_2_ evolution is significantly inhibited ([66], Figure 2b). The CO cultivation condition does not affect 1,2-propanediol formation, but less H_2_ is produced and the remaining reduced electron-carriers are now recycled by the formation of lactate and ethanol, consequently leading to a decrease in the acetate level. These findings suggest that, based on the substrate specificity of the lactate dehydrogenase (NADH, [57]) and the ethanol dehydrogenase(s) (NADPH, [57]), electron exchange between the Fd_red_ and NAD(P)^+^ is required. However, no genes have been identified in *C. saccharolyticus* coding for an enzyme capable of catalyzing such a reaction [33,60]. Transcript levels of the genes from the gene cluster containing both the L-rhamnose isomerase and L-rhamnulose-1-phosphate aldolase are highly upregulated during growth on rhamnose (Figure 3) [33]. Although the function of the other genes in the cluster with respect to rhamnose catabolism remains unclear, the absence of such gene cluster and the rhamnose kinase gene from the genome of other *Caldicellulosiruptor* species, strongly correlates with their inability of rhamnose degradation (Table 1). This difference in rhamnose degrading capability between *Caldicellulosiruptor* species reflects the open nature of the *Caldicellulosiruptor* pan genome [29].

**Table 1:** Overview of *Caldicellulosiruptor* species which are able to grow on rhamnose. There is a correlation between the ability to grow on rhamnose and the presence of gene clusters orthologous to the rhamnose associated gene clusters identified in *C. saccharolyticus* (Figure 3). nt, not tested for growth on rhamnose. (+), able to grow of rhamnose/gene cluster is present in genome. (−), no growth observed on rhamnose/gene cluster is not present in genome. ns, not sequenced.

Strain	Growth on Rhamnose	Gene cluster Csac_0865-76	Gene cluster Csac_0989-90	Reference
*C. saccharolyticus*	+	+	+	[13]
*C. bescii*	+	+	+	[28]
*C. owensensis*	+	+	+	[21]
*C. obsidiansis*	nt	+	+	[20]
*C. kronotskyensis*	nt	+	+	[22]
*C. hydrothermalis*	nt	+	+	[22]
*C. kristjanssonii*	−	−	−	[17]
*C. lactoaceticus*	−	−	−	[23]
*C. acetigenus*	nt	ns	ns	[26]

### 4.3. Involvement of Pyrophosphate in the Energy Metabolism

From a bioenergetics point of view, glucose fermentation is optimal when acetate is the only end product, because an additional ATP is generated during the final acetate-forming step (Figure 1). When it is assumed that the ABC-transporter mediated substrate uptake requires 1 ATP, overall ATP yields become 1.5 ATP per acetate versus only 0.5 ATP per lactate or ethanol. However, ATP yields might even be higher when the involvement of pyrophosphate (PP_i_) as an energy carrier is considered.

PP_i_ is a by-product of biosynthesis reactions like DNA and RNA synthesis or is generated when the amino acids are coupled to their tRNAs during protein synthesis [69]. Since these reactions are close to equilibrium accumulation of PP_i_ is believed to have an inhibitory effect on growth, and only the effective removal of PP_i_ drives these biosynthetic reactions forward [70]. When PP_i_ is hydrolysed to P_i_by a cytosolic inorganic pyrophosphatase (PPase), the free energy just dissipates as heat. *C. saccharolyticus* does not contain a cytosolic PPase but possesses a membrane bound H^+^-translocating PPase [71], which allows the free energy released upon PP_i_ hydrolysis to be preserved as a proton motive force. The high-energy phosphate bond of PP_i_ can also be used for the phosphorylation of fructose 6-phosphate, catalyzed by a PP_i_-dependent phosphofructokinase (Figure 1).

Furthermore, PP_i_ is consumed during the catabolic conversion of phosphoenolpyruvate to pyruvate, catalyzed by pyruvate phosphate dikinase (PPDK). Such a catabolic role for PPDK was proposed based on the increase in transcript level of the *ppdk* gene under increased glycolytic fluxes [33,71]. Altogether, the use of PP_i_ as an energy donor could be a way for the organism to deal with the relative low ATP yields which are usually associated with fermentation [72].

### 4.4. Mechanism Involved in Mixed Acid Fermentation

H_2_ has been reported as a growth inhibitor for *C. saccharolyticus* [16,73] and a critical dissolved H_2_ concentration, which leads to the complete inhibition of growth, of 2.2 mmol/L has been determined for controlled batch cultivations [74]. In addition, an elevated P_H2_ has been shown to cause a switch in the fermentation profile, leading to increased formation of ethanol and lactate, in both controlled batch and chemostat cultivations [12,60,74,75]. Willquist *et al.* reported that in controlled batch fermentations the initiation of lactate formation coincided with an increment in both the internal NADH/NAD^+^ ratio and the P_H2_ of the system [57,75].

An increase of the overall carbon flux through glycolysis results in a higher NADH production rate. To maintain a constant NADH/NAD^+^ ratio a subsequently higher H_2_ formation rate is required. However, when the H_2_ formation rate (volumetric H_2_ production rate) exceeds the H_2_ liquid to gas mass transfer rate, H_2_ will accumulate in the liquid phase. In some cases this can even lead to super saturation of the liquid, meaning that the dissolved H_2_ concentration exceeds the maximal theoretical H_2_ solubility [74,76]. Such high dissolved H_2_ levels inhibit H_2_ formation and presumably cause an increase in the NADH/NAD^+^ ratio. An increased NADH/NAD^+^ ratio has been shown to have an inhibitory effect on GAPDH activity thus limiting the glycolytic flux [75] and consequently leads to a decrease in substrate consumption and growth rate. A switch to lactate formation alleviates the inhibitory effect of an increased NADH/NAD^+^ ratio by causing a decrease of the NADH/NAD^+^ ratio through the reduction of pyruvate.

**Figure 4:**
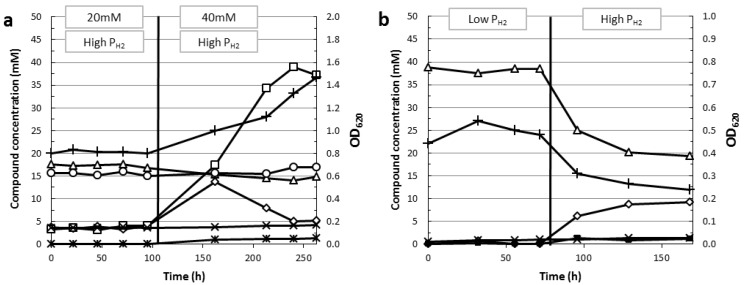
Fermentation profile of *C. saccharolyticus* (**a**) grown in a chemostat under high H_2_ partial pressure at two different glucose concentrations (see Section 4.4), (**b**) grown in a chemostat without NH_4_^+^ in the medium under low and high H_2_ partial pressure (see Section 6.4). The switch from 20 mM to 40 mM glucose containing-medium and the switch from low to high H_2_ partial pressure cultivation condition is indicated by a vertical line. Acetate (open triangles), lactate (open squares), ethanol (crosses), CO_2_ (open circles), oxaloacetate (asterisks), glucose out (open diamonds) and CDW (plus sign). Chemostat cultivation parameters (3 L reactor, 1 L working volume, pH = 7.0 (NaOH), temp 70 °C, D = 0.1 h^−1^, low P_H2_ [sparging (4 L/h) with N_2_ gas and stirring speed = 250 rpm]), high P_H2_ [headspace flushing (4 L/h) with H_2_ gas and stirring speed = 50 rpm]).

A clear switch to lactate formation caused by an increase in glycolytic flux can be observed for *C. saccharolyticus* grown in a chemostat under high P_H2_ ([77], Figure 4a). When the glucose load is increased from 20 mM to 40 mM, end-product formation is switched from mainly acetate to mainly lactate, respectively. After the switch to 40 mM glucose some adaptation time is required before a new steady state is achieved. During this adaptation period substantially higher amounts of glucose were detected in the culture effluent, probably indicating an inhibition of the glycolytic flux at the level of GAPDH. When a new steady state was achieved, residual glucose was only slightly higher with respect to the 20 mM condition. As a consequence of the increase in glycolytic flux, the lactate concentration dramatically increased while the acetate concentration hardly changed, indicating that during both substrate loads a similar volumetric H_2_ productivity was maintained. These data indicate that under these conditions the organism is not capable of dealing with the increased glycolytic flux by increasing its H_2_ productivity, but requires a switch to lactate formation. Although oxaloacetate formation was discernible under a 20 mM glucose load, significantly higher and therefore quantifiable levels of oxaloacetate were detected under the 40 mM glucose load condition. This observation together with the increased flux towards lactate suggests that in the newly achieved steady state, a bottleneck exists at the level of pyruvate.

Ethanol formation can also serve as reductant sink in *C. saccharolyticus*. For some chemostat cultivation conditions a decrease in H_2_ yield is only associated with ethanol formation and not with lactate formation [56,60,75]. These conditions concern low substrate loads, and the observed increase in ethanol formation was triggered by an increase of the dilution rate [56,75] or a change in mode of gas flushing [60], both potentially generating a moderate increase in the H_2_ level of the system.

### 4.5. Regulation of Reductant Disposal Pathways

**Figure 5:**
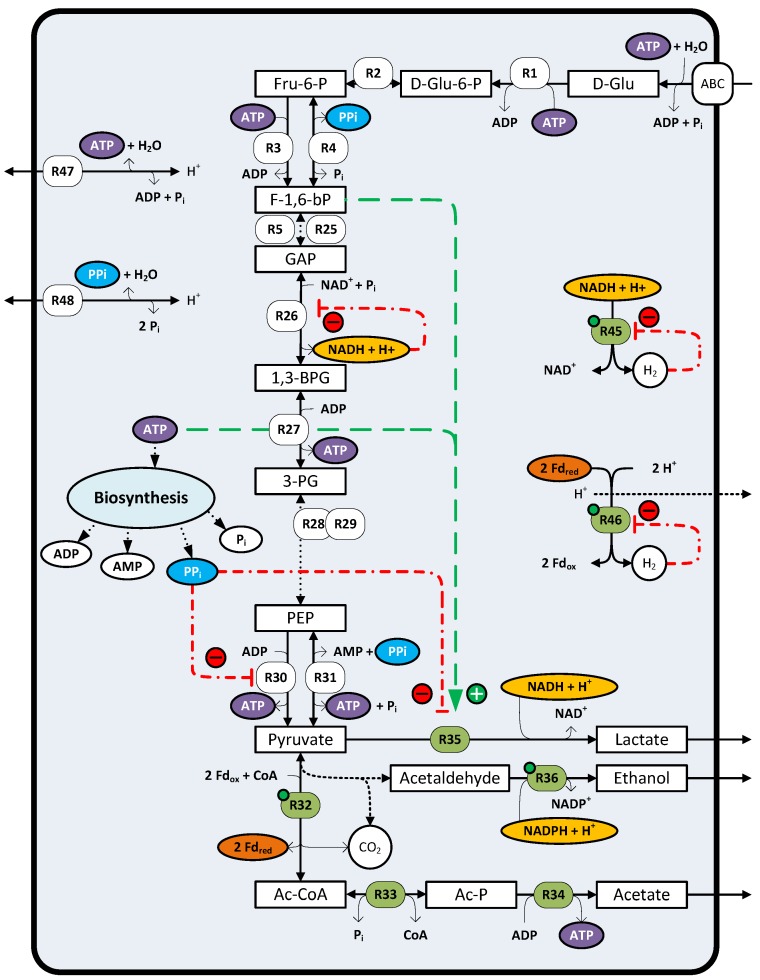
Overview of the regulatory mechanisms involved in the central metabolism of *Caldicellulosiruptor saccharolyticus*. The abbreviations of the compounds and reactions (circled numbers) are given in the legend of Figure 1. For the enzyme reactions given in green (circled numbers, green) the encoding genes are upregulated under increased P_H2_ [60]. Those which are under the control of the REX transcriptional regulator are marked with a green dot. H_2_ inhibits its own formation (**R45**, **R46**) and accumulation of NADH inhibits glyceraldehyde-3-phosphate dehydrogenase (**R26**). PP_i_, a by-product of biosynthesis, acts as an inhibitor of both pyruvate kinase (**R30**) and lactate dehydrogenase (**R35**) activity. Both ATP and F-1,6-bP are activators of lactate dehydrogenase (**R35**) activity.

Hydrogen, ethanol and lactate formation are the main routes for reductant disposal in *C. saccharolyticus*. The gene expression of the hydrogenases and both alcohol dehydrogenases involved in ethanol formation are proposed to be under the control of the NADH/NAD^+^ sensitive transcriptional regulator Rex [60] (Figure 5). Cultivation of *C. saccharolyticus* under high P_H2_ conditions was shown to lead to an upregulation of both the hydrogenases and alcohol dehydrogenases [60]. In addition, the *ldh* transcript level is also upregulated under high P_H2_ conditions, but in silico analysis of the *ldh* promoter region did not reveal a likely Rex operator binding sequence [60]. Thus, the exact regulatory mechanism triggering *ldh* transcription remains therefore unclear. Nonetheless, lactate dehydrogenase activity has been shown to be regulated at the enzyme level, with fructose 1,6-bisphosphate and ATP acting as allosteric activators and both NAD^+^ and PP_i_ as competitive inhibitors [57]. Under high P_H2_ cultivation conditions, enzyme activity assays revealed increased LDH activity with respect to low P_H2_ conditions [57,75]. A hampered glycolytic flux at the level of GAPDH, potentially triggered by NADH build-up, might lead to the accumulation of fructose 1,6-bisphosphate. In turn, the accumulated level of fructose 1,6-bisphosphate could stimulate lactate formation (Figure 5), explaining the switch to lactate under high P_H2_ at the enzyme level.

Interestingly, LDH enzyme activity can also be measured under non-lactate producing conditions, which suggest that additional factors control lactate formation [57,75]. Low levels of lactate formation can be observed at the end of growth during the transition to stationary phase for cultures grown in controlled batch systems [55,57,75,78,79]. This initiation of the lactate formation coincided with a relative increase in ATP levels and a relative decrease in PP_i_ levels [57,71]. These observed changes in ATP and PP_i_ levels could release the inhibitory effect of PP_i_ and stimulate LDH activity [57] (Figure 5). For this latter mode of LDH activity regulation, the lactate formation is proposed to be coupled to the energy metabolism. Accordingly, lactate formation is prevented during exponential growth, which is associated with a high anabolic activity and high PP_i_ levels. Whereas, during the transition to stationary phase, which is associated with low anabolic activity and relative low PP_i_ levels, lactate formation inhibition is alleviated [12,57].

## 5. Thermodynamic Considerations of Glucose Conversion and H_2_ Formation

For *C. saccharolyticus* an elevated hydrogen concentration has been shown to affect fermentation performance, leading to a mixed acid fermentation [60,73,74,75]. These observed changes in the fermentation profile as a function of the H_2_ concentration can be conceptually explained by considering the thermodynamics of the reactions leading to H_2_ formation [9,10]. The Gibbs energy (ΔG') for a specific reaction can be calculated from the standard Gibbs energy (ΔG^0^') and the reactant concentrations by the following relation:

ΔG' = ΔG^0^' + RT ln([C]^c^[D]^d^/[A]^a^[B]^b^) (4)

where ΔG^0^' is the standard Gibbs energy (J/mol, 1 mol concentration of all reactants, at a neutral pH and at a specific temperature), R is the gas constant (J/mol*K); T is the temperature (K), A and B are the substrate concentrations with respective stoichiometric reaction coefficients a, b; and C and D are the reaction products with respective stoichiometric reaction coefficients c, d.

When H_2_ formation occurs in the aqueous phase H_2_ supersaturation can occur. For instance, over-saturation of 12 to 34 times the equilibrium concentration has been reported for *C. saccharolyticus* cultivations [74]. This indicates that estimating the dissolved H_2_ concentration from the measured H_2_ partial pressure by using the equilibrium constant does not always give an accurate representation of the state of the system. So H_2_(aq), instead of the H_2_ partial pressure (P_H2_), should be used as a parameter when investigating the effect of H_2_ on the metabolism. Therefore, all Gibbs energy calculations discussed herein were performed using the Δ_f_G^0^ of the dissolved H_2_ concentration (H_2_(aq)).

**Figure 6:**
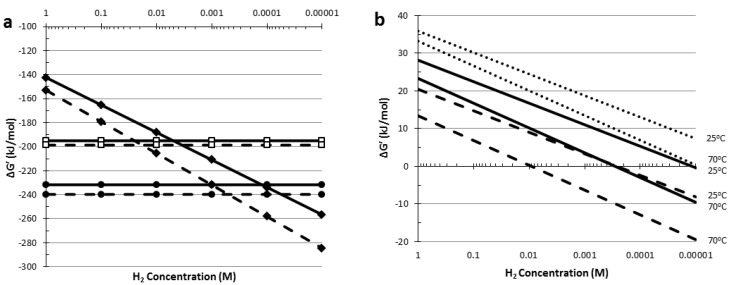
Effect of the H_2_ concentration on the Gibbs energy change of reactions involved in H_2_ formation: (**a**) ΔG' of the complete oxidation of 1 mol of glucose to acetate (closed diamonds), lactate (open squares) and ethanol (closed circles) at 25 °C (solid lines) and 70 °C (dashed lines); (**b**) ΔG' of H_2_ formation from NADH (dotted line), reduced ferredoxin (dashed line) and via the bifurcating system (50% NADH and 50% Fd_red_) (solid line) at 25 °C and 70 °C. Values were calculated from data presented in ([80,81,82,83,84]).

Under standard conditions (25 °C), acetate formation (Equation 1, ΔG^0^' = −142.6 kJ/reaction) is energetically less favorable then lactate (Equation 3, ΔG^0^' = −195.0 kJ/reaction) and ethanol (Equation 2, ΔG^0^' = −231.8 kJ/reaction) formation. The Gibbs energy (ΔG') for the complete conversion of glucose to acetate depends, however, on the H_2_ concentration (Equation 1), whereas the ΔG' for both ethanol and lactate formation from glucose is independent of the H_2_ concentration (Equations 2 and 3, respectively). This means that the complete oxidation of glucose to acetate becomes energetically more favorable when the dissolved H_2_ concentration is lowered. For example, acetate formation becomes energetically favorable compared to lactate or ethanol formation when the dissolved H_2_ concentration drops below 5.0 or 0.12 mM, respectively (Figure 6a, supplementary data S1).

However, considering the Gibbs energies of the overall conversions can be misleading, as the thermodynamics of the involved partial reactions can be less favorable. Acetate formation is inevitably linked to H_2_ formation and the ΔG^0^' of the redox reaction coupling the reoxidation of the reduced electron carriers NADH or Fd_red_ to proton reduction are endergonic under standard conditions (Figure 6b, supplementary data S2). Decreasing the dissolved H_2_ concentration will lower the ΔG' of these H_2_ forming reactions, but an extremely low H_2_ concentration is required before H_2_ formation from NADH becomes exergonic (0.5 µM) (Fd_red_, 0.2 mM) (Figure 6b). Therefore, at elevated H_2_ concentrations this partial reaction raises a thermodynamic barrier. NADH dependent pyruvate reduction to lactate, on the other hand, has a negative Gibbs energy (ΔG^0^' = −25.0 kJ/reaction) and is independent of the dissolved H_2_ concentration. This means that under certain conditions lactate formation is more feasible, despite the more negative ΔG' for acetate formation compared to lactate formation (Equations 1 and 3), simply because H_2_ formation from NADH is energetically unfavorable.

As explained, the thermodynamic barrier associated with H_2_ formation from NADH can be lowered by decreasing the dissolved H_2_ concentrations, moreover this barrier may also be tackled by an additional input of energy. The latter can be achieved by reverse electron transport catalyzed by an NADH: ferredoxin oxidoreductase, where the transfer of electrons from NADH to Fd_red_ is coupled to a proton or ion gradient [85]. Produced Fd_red_ can subsequently be used for H_2_ formation. Alternatively, the energetically more favourable oxidation of Fd_red_ can be used to push the less favourable formation of H_2_ from NADH in a bifurcating system (Figure 6b). Such a bifurcating function has been identified for *T. maritima* and was, based on protein sequences, also attributed to the Fe-only hydrogenase (*hyd*) of *C. saccharolyticus* [86]. However, the gene-arrangement of the Fe-only hydrogenase (*hyd*) in *C. saccharolyticus* is identical to that of *Thermoanaerobacter tengcongensis* [33], for which a NADH-dependent hydrogenase activity has been demonstrated [64], thus arguing against a bifurcating systems in *C. saccharolyticus*.

In general, H_2_ formation is thermodynamically more favorable at elevated temperatures because (i) ΔG^0^' values of the involved reactions are lower at increased temperature, and (ii) the RT coefficient in Equation 4 is temperature dependent, thus enhancing the effect of a decreased H_2_ concentration (Figure 6a,b). Overall, thermophilic organisms have been shown to be able to produce H_2_ at higher yields compared to mesophilic organisms. For thermophiles yields approaching the theoretical limit of 4 H_2_ per hexose [83] have been reported, while for mesophiles H_2_ yields generally do not exceed 2 H_2_ per hexose [9,11,12]. These higher yields reflect the indicated thermodynamic advantage but also the lower diversity of fermentation end products observed for those thermophiles. For a specific organism the diversity of available electron acceptors, and formed end products, depends on the metabolic capabilities of the organism. Whether a specific pathway is operational depends on the regulation of that pathway at the transcription or translation level, but also depends on the kinetic properties and regulation of the enzyme activities of the specific enzymatic steps of that pathway.

## 6. Factors Limiting H_2_ Formation

With an eye to the potential use of complex biomass for H_2_ production a multitude of fermentability studies have been performed using *C. saccharolyticus*. An overview of the literature related to fermentability studies on either crop-based feedstock or industrial waste stream derived biomass is given in Table 2. Those investigations mainly focus on the fermentability of various complex substrates, associated H_2_ formation and the effect of pretreatment on substrate accessibility and growth, overall demonstrating the bacterium’s broad hydrolytic capacity.

**Table 2:** Literature overview of fermentability studies on *C. saccharolyticus* using crop-based feedstock or industrial waste stream derived biomass. * Extraction methods and mechanical pre-treatments were excluded in this overview, biological pre-treatment indicated pre-treatment by pre-incubation with *Bacillus amyloliquefaciens*; ** Multiple, different pre-treatments used; B, batch cultivation; CB, controlled batch cultivation; ^$^ Minimal and maximal reported H_2_ yields are given.

Reference	Substrate	Pre-treatment *	Cultivation method	H_2_ yields ^$^/Remarks
[87]	Wheat grains	Enzymatic	B	
	Wheat straw	Acid/Enzymatic	B	
[88]	Barley straw	Acid/Enzymatic **	B	
[29]	Crystalline cellulose	-	B	Proteome data
	Birchwood xylan	-	B	
	Switchgrass	Acid	B	
	Whatman no. 1 filterpaper	-	B	
[89]	Wheat straw	Acid/Enzymatic	B	
	Barley straw	Acid/Enzymatic	B	
	Corn stalk	Acid/Enzymatic	B	
	Corn cob	Acid/Enzymatic	B	
[36]	Poplar	-	B	Microarray data
	Switchgrass	Acid	B	Microarray data
[90]	Beet Molasses	-	CB	0.9–4.2
[91]	Potato steam peels	Enzymatic	CB	1.7–3.4
	Potato steam peels	-	CB	1.1–3.5
[92]	Filter paper	-	B	
	Wheat straw	Biological	B	
	*Silphium perfoliatum* leaves	Biological	B	
	*Maize* leaves	Biological	B	
	Sugar cane bagasse	Biological	B	
	Sweet sorghum whole plant	Biological	B	
[93]	Sugar beet	-	CB	3.0
[94]	Sweet sorghum bagasse	Alkaline/Enzymatic **	B/CB	1.3–2.6
[55]	Carrot pulp	Enzymatic	CB	1.3–2.8
	Carrot pulp	-	CB	
[35]	Crystalline cellulose	-	B	Proteome data
	Cellobiose	-	B	Microarray data
[95]	Switchgrass	-	B	
	Poplar	-	B	
[53]	Xylan	-	B	Microarray data
	Xyloglucan	-	B	Microarray data
	Xyloglucan-oligosaccharides	-	B	Microarray data
[96]	Barley straw	Acid/Enzymatic	B	
	Corn stalk	Acid/Enzymatic	B	
	Barley grain	Enzymatic	B	
	Corn grain	Enzymatic	B	
	Sugar beet	-	B	
[97]	Sweet sorghum plant	-	B	
	Sweet sorghum juice	-	B	
	Dry sugarcane bagasse	-	B	
	Wheat straw	-	B	
	*Maize* leaves	-	B	
	*Maize* leaves	Biological	B	
	*Silphium trifoliatum* leaves	-	B	
[54]	*Miscanthus giganteus*	Alkaline/Enzymatic	B/CB	2.4–3.4
[52]	Agarose	-	B	With different support matrixes
	Alginic acid	-	B	
	Pine wood shavings	-	B	
[98]	Jerusalem artichoke	-	B	Co-fermentation with natural biogas-producing consortia
	Fresh waste water sludge	-	B	
	Pig manure slurry	-	B	
[78]	Paper sludge	Acid/Enzymatic	CB	
[99]	Paper sludge	Acid/Enzymatic	B	

On the other hand, growth experiments on pure sugar substrates can be used to investigate the specific response associated with a certain substrate or to examine specific pathways involved in the metabolism of a substrate. Table 3 gives an overview of the literature related to growth experiments on pure sugars and pure sugar mixes, including the determined H_2_ yields and H_2_ productivities. The currently available fermentation data are discussed here to highlight the different factors limiting H_2_ formation during the fermentative H_2_ production by *C. saccharolyticus*.

**Table 3:** Literature overview of fermentation studies on *C. saccharolyticus* grown on pure sugar substrates. * Yields in mol H_2_/mol hexose; nd, not determined; ** For batch cultivations the maximal productivity is given; nd, not determined; B, batch cultivation; and CB, controlled batch cultivation; Chem, chemostat cultivations.

Reference	Substrate	Substrate	H_2_ Yield *	Productivity **	Cultivation method	Dilution rate (h^−1^)	Remarks
		load (g/L)		(mmol/(L*h))			
[100]	Glucose	10	3.0	20.0 mol/(g*h)	CB		
		10	3.4	23.6 mol/(g*h)	CB		No YE in medium
		4	3.5	10.1 mol/(g*h)	Chem	0.05	
		4	3.5	10.4 mol/(g*h)	Chem	0.05	No YE in medium
[75]	Glucose	5	3.5	5.2	Chem	0.05	
		5	2.9	11.0	Chem	0.15	Residual glucose (3 mM)
		5	1.8	2.5	Chem	0.05	no sparging, open gas outlet
		5	wash out	wash out	Chem	0.15	no sparging, open gas outlet
[101]	Glucose	5	nd	nd	B		Extracellular proteome
[91]	Glucose	10	3.4	12.0	CB		
		31	2.8	12.9	CB		Residual glucose
[93]	Sucrose	10	2.9	7.1	CB		
[94]	Glucose/Xylose/Sucrose	10	3.2	10.7	CB		Sugar mix
	(6:2.5:1.5, w/w/w)	20	2.8	9.4	CB		Sugar mix
[55]	Glucose	10	3.2	11.2	CB		
		20	3.4	12.2	CB		
	Fructose	10	2.6	13.2	CB		
		20	2.4	13.4	CB		
	Glucose/Fructose	10	3.0	13.2	CB		Sugar mix
	(7:3, w/w)	20	2.6	12.2	CB		Sugar mix
[35]	Xylose	5	nd	nd	B		Proteome data
	Glucose	5	nd	nd	B		Proteome data
[50]	Glucose	5	nd	nd	B		Extracellular proteome
[79]	Sucrose	4	2.7	23.0	CB		
		4	3.1	11.8	CB		CO_2_ sparging
	Glucose	4	3.0	20.0	CB		
		4	2.7	12.0	CB		CO_2_ sparging
[53]	Glucose	0.5	nd	nd	B		Microarray data
	Mannose	0.5	nd	nd	B		Microarray data
	Arabinose	0.5	nd	nd	B		Microarray data
	Xylose	0.5	nd	nd	B		Microarray data
	Fructose	0.5	nd	nd	B		Microarray data
	Galactose	0.5	nd	nd	B		Microarray data
	mix (0.5 g/L each)	3	nd	nd	B		Microarray data, Sugar mix
[54]	Glucose/Xylose	10	3.4	12.0	CB		Sugar mix
	(7:3 w/w)	14	3.3	10.1	CB		Sugar mix
		28	2.4	9.7	CB		Sugar mix
[102]	Sucrose	10.3	2.8	22.0	Trickle bed	0.2–0.3	400 L, non-axenic fermentation
[33]	Glucose	4	nd	nd	CB		Microarray data
	Xylose	4	nd	nd	CB		Microarray data
	Rhamnose	4	nd	nd	CB		Microarray data
	Glucose/Xylose (1:1, w/w)	4	nd	nd	CB		Microarray data
[56]	Glucose	4.4	3.3	4.2	Chem	0.05	
		4.4	3.6	8.9	Chem	0.10	
		4.4	2.9	9.5	Chem	0.15	Residual glucose (3.3 mM)
		4.4	2.9	9.1	Chem	0.20	Residual glucose (8.9 mM)
		4.4	3.1	11.0	Chem	0.30	Residual glucose (12.4 mM)
		4.4	3.0	12.4	Chem	0.35	Residual glucose (12.7 mM)
		1.9	4.0	4.0	Chem	0.09	
		1.9	3.3	9.9	Chem	0.30	Residual glucose (0.6 mM)
		4.1	3.5	7.7	Chem	0.09	
		4.1	3.1	11.6	Chem	0.30	Residual glucose (11.9 mM)
[78]	Glucose	10	2.5	10.7	CB		
	Xylose	10	2.7	11.3	CB		
	Glucose/Xylose (11:3, w/w)	8.4	2.4	9.2	CB		Sugar mix
[103]	Sucrose	10	3.3	8.4	CB		

### 6.1. Comparison between Hydrolysates and Pure Sugar Mixtures

Studies on biomass hydrolysates and mono-saccharide mixtures, mimicking the biomass hydrolysates, showed that, while at low substrate loads fermentation performances were comparable, at higher substrate loads H_2_ yields were higher on the mixed mono-saccharides compared to the biomass hydrolysates. The difference in yields was caused by a shift to lactate formation during the growth on high substrate load hydrolysates. Interestingly, for the higher substrate concentrations, the total sugar consumption was higher during growth on hydrolysates compared to growth on sugar mixtures [55,91]. For *C. saccharolyticus*, grown on carrot pulp hydrolysate (20 g/L), a lower cumulative H_2_ production was found, compared to growth on a glucose/fructose mixture (20 g/L), while a relatively higher maximal H_2_ productivity was observed for the hydrolysate compared to the sugar mixture. During the growth on carrot pulp hydrolysates the relative higher H_2_ productivity preceded the switch to lactate formation [55]. These results demonstrate the relation between high H_2_ productivity, lactate formation and an overall low H_2_ yield. However, these described phenomena were not observed for fermentations on *Miscanthus* hydrolysates. For each tested substrate load (10, 14 and 28 g/L) fermentation performance during growth on the *Miscanthus* hydrolysate was similar to the glucose/xylose sugar mix and only moderate levels of lactate were formed even at high substrate loads [54]. The differences in H_2_ production characteristics between the discussed hydrolysates might be related to the difference in sugar composition of the hydrolysates or the differences in pretreatment applied prior to hydrolysis.

In general, biomass derived hydrolysates might contain substances which negatively affect growth or fermentation performance. For example, the dilute-acid pretreatment of lignocellulosic biomass releases undesirable inhibiting compounds like 5-hydroxymethylfurural (HMF), furfural, phenolic compounds and acetate [89] and a growth inhibition of 50% was reported for *C. saccharolyticus* in the presence of 1–2 g/L HMF or furfural [54].

### 6.2. Incomplete Substrate Conversion

For chemostat cultivation on glucose (4.4 and 5 g/L) it was shown that a dilution rate (D) exceeding 0.1 h^−1^ gave rise to an incomplete substrate conversion but also to a lower H_2_ yield. The concomitant decrease in both biomass level and H_2_ yield caused the volumetric H_2_ productivity (mmol/L*h) to level off at higher dilution rates [56,75]. The observed lower H_2_ yield reflects the shift in end product formation, from mainly acetate at a low dilution rate of 0.05 h^−1^ to a mix of acetate and ethanol at a D = 0.15 h^−1^ [75]. Interestingly, no lactate was produced during these chemostat cultivations [56,75]. The observed incomplete substrate conversion indicated that another factor was limiting under those conditions. Indeed, increasing the yeast extract to glucose ratio, from 0.25 to 1 g/g, resulted in the almost complete consumption of glucose and also a doubling of cell density (D = 0.3 h^−1^), which led to an increase in volumetric H_2_ productivity (20 mmol/L*h). The H_2_ yield was, however, not affected [56]. This finding indicated that these observed changes in H_2_ yields were a function of the growth rate and did not depend on the substrate conversion efficiency.

### 6.3. End Product Inhibition and Osmotolerance

Incomplete substrate conversions can also be observed in controlled batch fermentations with high initial substrate levels. Growth on 10 g/L of both glucose and fructose resulted in complete substrate consumption, while higher initial substrate levels of glucose (20 and 31 g/L) and fructose (20 g/L) resulted in incomplete conversions [55,91]. Similar observations were done for different sugar mixtures [54,55,94]. These incomplete conversions were attributed to the inhibitory effect of accumulating organic acids like acetate or lactate. Inhibition experiments showed that an acetate concentration of 200 mM and higher prevented acid production by *C. saccharolyticus* grown on glucose (10 g/L) in batch [91], which is in line with the earlier findings of van Niel *et al*., who observed critical sodium acetate and potassium acetate concentrations of 192 mM and 206 mM, respectively, for batch growth on sucrose [73]. However, similar inhibitory effects were observed for NaCl and KCl, with critical concentration of 216 mM and 250 mM respectively [73], suggesting that increased osmolarity is the cause of inhibition and not the end products per se. This relative low osmotolerance in comparison to marine organisms, like *Thermotoga neapolitana* [91], probably reflects the terrestrial origin of *C. saccharolyticus*. Ljunggren *et al*. designed a kinetic model for the growth of *C. saccharolyticus* incorporating the inhibitory effect of a high osmolarity and determined a critical osmolarity (no growth) in the range of 270 to 290 mM. They also showed that osmolarity is of minor influence on fermentations with low initial glucose levels [74]. This low tolerance to osmotic pressure also prevents the application of CO_2_ as a cheaper and more convenient stripping gas than N_2_. The use of CO_2_ as stripping gas during *C. saccharolyticus* cultivations negatively affects growth rate and hydrogen productivity. CO_2_ sparging led to a higher dissolved CO_2_ concentration, which required addition of extra base to maintain a constant pH, overall leading to an increase in osmotic pressure [79].

### 6.4. Medium Requirements

Controlled batch cultivations were used to investigate the influence of NH_4_^+^ on the performance of *C. saccharolyticus* grown on molasses. These experiments revealed that the omission of NH_4_^+^ gave rise to a higher H_2_ yield and maximal H_2_ productivity [90]. Although *C. saccharolyticus* is able to grow on a medium without NH_4_^+^, containing only YE as a nitrogen source, it becomes very sensitive to changes in P_H2_ ([77], Figure 4b). Chemostat cultivations showed complete glucose (20.7 mM) consumption and high acetate yields (1.87 ± 0.02 mol/mol) under low P_H2_. However, when the cultivation condition changed to a high P_H2_ a new steady state could be achieved, but substrate consumption was incomplete (55%) and acetate yields decreased (1.68 ± 0.01 mol/mol).

Omission of yeast extract (YE) during growth of *C. saccharolyticus* on molasses did not affect the H_2_ yield but led to a lower volumetric H_2_ productivity [90]. Similar observations were made for controlled batch fermentations on glucose, where the absence of YE did not affect the H_2_ and biomass yields [100]. Contrary to the molasses study the volumetric productivity was not affected [100] but this might be due to the lower substrate load used. *C. saccharolyticus* is able to grow on a defined minimal medium with additional vitamins, but without additional amino acids [100]. Growth in the absence of YE helps to reduce the production costs. However, increased biomass levels and especially growth on high substrate loads might augment medium requirements. So fine-tuning of the medium composition with respect to the specific substrate and substrate load is required.

## 7. Future Prospects for Improving Biohydrogen Production

### 7.1. Improving H_2_ Yields and H_2_ Productivity

*C. saccharolyticus* has many properties that make it an excellent candidate for biohydrogen production via dark fermentation. However, for biohydrogen production to become economically feasible major improvements should be made with respect to the H_2_ productivity [104,105,106]. Productivity is maximized by improving the substrate consumption rate but also the H_2_ yield.

*C. saccharolyticus* is able to produce H_2_ close to the theoretical maximum of 4 H_2_ per hexose. In this respect, it is important to realize that the theoretical yield refers to the pure catabolic component of glucose conversion. The glucose used for anabolism should not be incorporated. Generally, this distinction is not considered in literature, and reported experimental data therefore reveal H_2_ yields lower than 4. For example, a yield of 3.5 H_2_ per consumed glucose has been reported by de Vrije *et al.* (chemostat cultivation with a 23.0 mM glucose load and a dilution rate of 0.1 h^−1^) [56]. According to their data 16% of the consumed glucose is used only for biomass formation indicating that only ~19.4 mM glucose was available for ATP generation. Given the reported H_2_ production this results in a theoretical conversion efficiency of 4.15 mol H_2_ per mol glucose, which approximates the theoretical maximum of 4 H_2_ per hexose as indicated by Thauer *et al*. [83]. These high yields are only achievable when the organism ferments the substrate solely to acetate. However, non-ideal growth conditions lead to a mixed fermentation profile (acetate, lactate and ethanol), and a consequently lower H_2_ yield. From the organism’s perspective switching to lactate or ethanol is profitable since it allows the organism to continue to grow under elevated P_H2_ conditions, albeit with a lower growth rate because ATP yields are lower under lactate and ethanol forming conditions. Obviously, the lower H_2_ yield under a mixed end-product fermentation, is not desirable from a biotechnological point of view. To maximize the H_2_ yield and productivity, the dissolved H_2_ concentration should be kept as low as possible, which requires an optimization of the liquid to mass transfer rate [74,76], which is mainly a matter of reactor design. In continuous stirred-tank reactor systems low dissolved H_2_ concentrations could be achieved by increasing the sparging rate [107,108] or the stirring speed [109,110]. In addition, reduction of internal reactor pressure [111,112,113] and enforced bubble formation [113,114] could potentially lead to a lower dissolved H_2_ concentration. Addition of zeolite particles, which enhances bubble formation, allowed a reduction of the N_2_ stripping rate from 5 L/(h*L) to 1 L/(h*L), without affecting the H_2_ productivity and H_2_ yield of *C. saccharolyticus*. N_2_ stripping could even be completely omitted when an internal reactor pressure of 0.3 bar was used, which sustained a similar fermentation performance compared to cultivation at atmospheric pressure (1 bar) using a N_2_ gas stripping rate of 5 L/(h*L) [113]. 

Most research on *C. saccharolyticus* has been performed in serum bottles and suspended continuous stirred-tank reactor systems, where only relatively low cell biomass levels can be achieved. Higher cell biomass levels would cause an increase in substrate consumption rates leading to an increase in H_2_ productivity when H_2_ yields are maintained. To realize higher biomass levels a deeper insight into growth limiting medium compounds should be acquired. Additionally, other reactor types, like a trickle bed reactor or a fluidized bed system might allow cell biomass accumulation. *C. saccharolyticus* could be cultivated in a non-axenic 400 L trickle bed reactor [102] out-competing other organisms, with H_2_ yields around 2.8 mol H_2_/mol hexose and a productivity of 22 mmol H_2_/L*h. These results showed that *C. saccharolyticus* can be used in a large scale non-sterile industrial setting.

Combining different organisms in a co-cultivation setup allows the exploitation of the hydrolytic capability of each individual species and could enhance the overall range of useable substrates. Batch co-cultivations of *C. saccharolyticus* with either *C. owensensis*, *C. kristjanssonii* or an enriched compost microflora were performed on a glucose-xylose mixture [115]. The co-cultivation with the enriched compost microflora resulted in a fast, simultaneous consumption of both glucose and xylose with a relatively high specific hydrogen production rate, but with a lower H_2_ yield [115]. A stable co-culture consisting of two closely related *Caldicellulosiruptor* species, *C. saccharolyticus* and *C. kristjanssonii*, could be established in a continuous cultivation system [116]. These findings demonstrate the possibility to create co-cultures for H_2_ formation and reveal an apparent synergistic effect of the strains, which lead to improved fermentation performances.

Overall H_2_ yields from biomass derived substrates can be increased when the dark fermentation is coupled to a second stage like electrohydrogenesis or photofermentation [117,118]. The former system uses a microbial electrolysis cell (MEC), in which electricity is used to convert acetate or other organic acids to hydrogen. In the latter case the main end product of the dark fermentation, acetate, is further converted by an anaerobic non-sulfur purple photosynthetic bacterium, forming a maximum of 4 mol H_2_ per mol acetate, giving an overall H_2_ yield of 12 mol H_2_ per mol glucose. The effluent of *C. saccharolyticus* has been successfully used as a feed for photofermentative growth and H_2_ production [90,119,120]. Alternatively, dark fermentation end-products H_2_ and acetate could serve as substrates for hydrogenothropic methanogens in a biogas generating system. The addition of *C. saccharolyticus* to natural biogas-producing consortia led to an improvement of biogas production and a stable co-cultivation could be maintained for several months [98].

### 7.2. Genetic Engineering of Caldicellulosiruptor Species

The first steps in the development of a genetic system for *Caldicellulosiruptor* species have been made. Chung *et al.* have shown that methylation with an endogenous unique α-class N4-Cytosine methyltransferase is required for transformation of DNA isolated from *E. coli* into *Caldicellulosiruptor bescii* [121]. Furthermore, an uracil auxotrophic *C. bescii* mutant strain was generated by a spontaneous deletion in the *pyrBCF* locus [121]. This nutritional deficiency was exploited as a selection marker of *C. bescii* transformants [121]. A similar strategy might be applied to develop a genetic system for the other members of this genus.

To improve the H_2_ producing capabilities of *C. saccharolyticus* or other *Caldicellulosiruptor* species metabolic engineering strategies could focus on improving the H_2_ yields. Additionally it could be aimed at altering intrinsic properties of *Caldicellulosiruptor* species limiting H_2_ productivity like the enhancement of their H_2_ tolerance or osmotolerance. For example, for industrial applications increased substrate concentrations are favored since it reduces the fresh water demand, thus reducing the overall costs and the environmental impact of the process. However, for *C. saccharolyticus* the maximum substrate load is limited by its sensitivity to osmotic stress [12,73,74].

With respect to the mixed acid fermentation, both lactate and ethanol formations lead to a lowered H_2_ yield. Because ethanol formation is not the major reductant sink under redox stress and in general is only produced at low levels, knocking out the alcohol dehydrogenase responsible for ethanol formation will probably not significantly alter the fermentation performance of *C. saccharolyticus*. However, lactate formation can be seen as the main mechanism to alleviate redox stress. Targeting the lactate formation pathway for complete knockout will probably make *C. saccharolyticus* less resilient to fluctuations in dissolved H_2_ concentration and is inadvisable.

Alternatively, one could alter glycolysis in such a way that substrate conversion is less energy efficient. So to generate the same amount of ATP, essential for biosynthesis and maintenance, a higher glycolytic flux to acetate is required. This would result in a higher H_2_ yield because the glycolytic flux to acetate is increased with respect to the carbon flux to biomass. A less energy efficient glycolysis can be achieved by eliminating some ATP generating steps from the central metabolic pathway. For example, exchanging the NADH-dependent GAPDH in *C. saccharolyticus* with the ferredoxin-dependent GAPOR, would decrease the overall ATP yield of glycolysis. In addition, since the H_2_ formation from the generated Fd_red_ is energetically more favourable than H_2_ formation from NADH, the organism would become less sensitive to increased H_2_ levels [9,12].

H_2_ yields on rhamnose and fucose could be increased if the carbon flux from the intermediate lactaldehyde is redirected via methylglyoxal to pyruvate, by the insertion of a lactaldehyde dehydrogenase and a methylglyoxal dehydrogenase. When rhamnose is completely oxidized to acetate, via this pathway, the H_2_ yield increases from 1 to 5 H_2_ per rhamnose. With respect to alternative substrates, glycerol could serve as a good substrate for H_2_ production because of the relative high reduced state of its carbon atoms. A maximum H_2_ yield of 3 mol/mol glycerol is achieved if glycerol is completely oxidized to acetate. So far, growth or co-consumption on/of glycerol has, however, not been observed for *C. saccharolyticus* [122].

## 8. Conclusions

The bacterium *Caldicellulosiruptor saccharolyticus* possesses several features that make it an excellent candidate for biological hydrogen production. With an optimal growth temperature of 70 °C *C. saccharolyticus* is one of the most thermophilic cellulose degrading organisms known to date. The organisms diverse inventory of endo- and exo-glycoside hydrolases allow it to degrade and grow on a variety of cellulose- and hemicellulose-containing biomass substrates. Some of these glycosidases are multi-domain proteins that contain both glycoside hydrolase domains and carbon binding modules, which facilitate the efficient degradation of recalcitrant plant polysaccharides into mono-, di- or oligo-saccharides. The high diversity of transport systems present in the genome confirm the broad substrate preferences of *C. saccharolyticus* and its ability to co-utilization hexoses and pentoses, without any signs of carbon catabolite repression, is a desirable trait for any consolidated bioprocess.

For *C. saccharolyticus* sugar substrates are primarily fermented to acetate, CO_2_ and H_2_, via the Embden-Meyerhof pathway. Typically, the fermentation of hexose and pentose lead to the generation of the reduced electron carriers NADH and Fd_red_ in a 1:1 ratio. These reduced electron carriers can be reoxidized during two distinct H_2_ generating steps, respectively catalyzed by an NADH-dependent cytosolic Fe-only hydrogenase (*hyd*) and the Fd_red_-dependent membrane-bound [NiFe] hydrogenase (*ech*). Alternatively, rhamnose catabolism is coupled to a different type of redox balancing, where the generated NADH is used for 1,2-propanediol formation and only the Fd_red_ is available for H_2_ formation.

Fermentation data reveal that *C. saccharolyticus* is capable of producing H_2_ with yields close to the theoretical limit of 4 H_2_ per hexose. However, under non-ideal conditions both ethanol and lactate formation act as alternative redox sinks, thus reducing H_2_ yields. All possible redox sinks, including the hydrogenases, are upregulated during cultivation under an increased partial hydrogen pressure. The mechanism underlying transcription of the lactate dehydrogenase gene remains elusive, but the transcription of the genes coding for both hydrogenases and the alcohol dehydrogenases, potentially involved in ethanol formation, seem to be under the control of an NADH/NAD^+^-sensing transcriptional regulator REX.

An increased intracellular NADH/NAD^+^ ratio, putatively caused by the inhibition of hydrogenase activity at elevated H_2_ levels, can hinder glycolysis at the level of glyceraldehyde-3-phosphate dehydrogenase, resulting in the inhibition of growth. Lactate formation serves as an alternative redox sink, alleviating redox stress. Lactate dehydrogenase activity is enhanced by the glycolytic intermediate fructose-1,6-bisphosphate but also modulated by the energy carriers ATP and pyrophosphate. The latter mechanism couples lactate formation to the energy metabolism, where lactate formation is inhibited during exponential growth and inhibition is alleviated during the transition to the stationary phase.

Overall, maintaining low dissolved H_2_ levels in the system appeared to be one of the most important factors for optimizing H_2_ production. In addition, improvements should be made with respect to the H_2_productivity and osmotolerance of the organism to allow biohydrogen production by *C. saccharolyticus* to become economically feasible.

## Supplementary Files

Supplementary File 1 Supplementary Table 1 (XLSX, 19 KB)

Supplementary File 2 Supplementary Table 2 (XLSX, 24 KB)
